# Obstructive sleep apnea knowledge and attitudes among recent medical graduates training in Ecuador

**DOI:** 10.1186/s40248-018-0117-8

**Published:** 2018-02-21

**Authors:** Iván Chérrez-Ojeda, Juan Carlos Calderón, Andrea Fernández García, Donna B. Jeffe, Ilka Santoro, Emanuel Vanegas, Annia Cherrez, José Cano, Freddy Betancourt, Daniel Simancas-Racines

**Affiliations:** 1 Universidad Espiritu Santo, Samborondon, Ecuador; 2 Respiralab Research Group, Respiralab, Guayaquil, Ecuador; 3 Department of Medicine, Washington University School of Medicine, St. Louis, MO USA; 4 Department of Medicine, University Federal of Sao Paulo, Sao Paulo, Brazil; 5 School of Medicine, University of Heidelberg, Heidelberg, Germany; 6 Centro de Investigación en Salud Pública y Epidemiologia Clínica, Facultad de Ciencias de la Salud Eugenio Espejo, Universidad Tecnológica Equinoccial, Quito, Ecuador

**Keywords:** Obstructive sleep apnea, Medical student, Knowledge, Physician, Questionnaire, OSAKA

## Abstract

**Background:**

We aimed to assess recent Latin American medical school graduates’ knowledge and attitudes about OSA and examine whether their knowledge and attitudes about OSA differed from practicing physicians.

**Methods:**

Recent medical graduates completed the Spanish translation of the OSA Knowledge and Attitudes (OSAKA) questionnaire at the 2013 national primary-care residency-placement meeting in Ecuador. The OSAKA includes 18 knowledge and five attitudinal items about OSA. We compared recent graduates’ data with data collected in 2010–2011 from practicing physicians using chi-square tests of associations among categorical variables and analysis of variance of differences in mean knowledge and attitude scores. Unadjusted logistic regression models tested the odds that recent graduates (vs. practicing physicians) answered each item correctly.

**Results:**

Of 265 recent graduates, 138 (52.1%) were male, and mean age was 25.9 years. Although mean knowledge was low overall, scores were lower for recent graduates than for the 367 practicing physicians (53.5% vs. 60.4%; *p* < 0.001). Practicing physicians were significantly more likely to answer specific items correctly with one exception—recent graduates were more likely to know that < 5 apneas-hypopneas/h is normal (OR 1.47, 1.03–2.07). Physicians in practice attributed greater importance to OSA as clinical disorder and the need for identifying patients with OSA; but recent graduates reported greater confidence in managing patients with OSA and CPAP.

**Conclusions:**

OSA-focused educational interventions during medical school should help to improve recent medical graduates’ abilities to diagnose and treat OSA. We recommend a greater number of hours of medical students’ exposure to sleep education.

## Background

Obstructive sleep apnea (OSA) is defined as repeated episodes of upper airway closure during sleep, resulting in oxy-hemoglobin desaturation and sleep fragmentation. This produces diurnal sleepiness and can lead to cognitive impairment and cardiovascular morbidity [1]. An estimated 2–4% of the middle-aged population is affected [2, 3]. OSA has been identified as an independent risk factor for hypertension, cardiovascular disease, abnormalities in glucose metabolism, depression and sleepiness-related accidents [4, 5]. Diagnoses can be made through full-night sleep studies in clinic or at home using polysomnography (PSG) or respiratory polygraph. OSA at any level of severity is reported to lead to impairment in patients’ quality of life [6]. Continuous positive airway pressure (CPAP) is the gold standard treatment [7]. There are medical morbidities, including new-onset cardiovascular disease and hypertension [8]. In Latin American countries, use of these treatments for OSA is limited, not only due to cost, but also to lack of physicians’ knowledge about OSA [9], which impedes diagnosis and treatment of the disease.

The low physicians’ knowledge and its clinical suspicion about OSA lead to under diagnosis [10, 11]. Few studies have examined whether length of physicians’ practice experience is associated with knowledge and attitudes about OSA, and its treatment [9, 12–15]. Recent studies in China and Saudi Arabia have assessed medical students’ knowledge about sleep disorders, but neither study specifically measured knowledge about OSA and its treatment [16, 17].

A recent study of Latin American general practitioners found no significant association between knowledge of OSA and number of years in practice [9], suggesting an overall lack of sleep medicine content in the medical school curriculum in Latin American countries over time. In Ecuador, Universities have 40 h of Respiratory Medicine curriculum and only 10% are dedicated to teach sleep apnea. The level of medical students’ knowledge of OSA at the time of graduation, therefore, can provide insight into their future medical practice in diagnosing OSA and prepare them to be alert when a patient presents with possible sleep apnea.

Thus, the objective of our present study was to assess the knowledge and attitudes of recent Latin American medical school graduates using a validated questionnaire. We compared these results with knowledge of General Physician in order to determinate if this knowledge is lower and if the reason could be an inadequate sleep education curriculum during medical school.

## Methods

### Study design

In 2013, we conducted an anonymous, cross-sectional survey of recent medical school graduates from both public and private universities in Ecuador as well as Ecuadorian students who attended foreign universities in Cuba and the Dominican Republic. All these universities have medical schools accredited by the government of Ecuador. In 2013, the surveys were administered during an annual, national meeting in Guayaquil for allocation of community service placement at primary care settings across Ecuador. Every year, about 1000 recent medical graduates attend this annual meeting. Community service is a requirement in Ecuador in order to obtain a professional license to practice. The institutional review board at the Kennedy Clinic at Guayaquil approved the study. Participants provided verbal consent to participate in the study before completing the questionnaire. No financial incentive was offered, and participation was voluntary.

### Study survey

We used the Spanish-translation of the OSAKA questionnaire [9] that was previously developed and validated in English in the USA [12, 13] to assess physicians’ knowledge and attitudes concerning the identification and management of patients with OSA. The OSAKA questionnaire includes 18 knowledge items and five questions related to attitudes about OSA. The knowledge items covered different OSA domains about epidemiology, pathophysiology, symptoms, diagnosis, and treatment.

Options for answers to knowledge questions were “true,” “false,” or “do not know,” which was scored as an incorrect response. Total knowledge scores were computed as the percentage of correct answers to the 18 knowledge questions and ranged from zero to 100%. Two attitude questions asked about importance of OSA, and responses were scored on a 5-point Likert scale, ranging from not important [1] to extremely important [5]. The other three attitude questions dealt with one’s confidence in diagnosing and treating patients with OSA, and responses were scored from strongly disagree [1] to strongly agree [5]. Mean scores were computed for each of the two attitude scales.

### Data analysis

All fully completed questionnaires were included in the analysis. Descriptive statistics were used to summarize answers to each question about knowledge (frequency and percentage answering correctly) and attitudes (mean and standard deviation [SD]). We merged data from our sample of recent medical school graduates with data collected in 2010–2011 for a study of practicing physicians in Latin American countries [9] to compare knowledge and attitudes for recent graduates vs. physicians already in practice. To help us target areas for sleep education curriculum design during medical school, we used chi-square tests to examine the proportions of different comparison groups who answered each knowledge item correctly: 1) recent graduates vs. physicians in practice, and for recent graduates only, students who graduated from private vs. public universities. Analyses of variance (ANOVAs) also were used to examine differences in the mean scores for knowledge and attitudes (importance of OSA and confidence) by each of these comparison groups. Unadjusted logistic regression models were used to determine odds of answering each item correctly comparing recent graduates with practicing physicians. Statistical tests were performed using SPSS version 19 (IBM SPSS, Inc., Chicago, IL, 2000). A two-tailed *P* < 0.05 was considered significant for all tests.

## Results

Out of 939 graduates who attended the meeting, 265 (28.2%) completed the entire survey. Most of the participants were male (52.1%) and graduated from public universities (80.2%); participants were 25.9 years old, on average (SD, 3.0).

### Knowledge

**Fig. 1:**
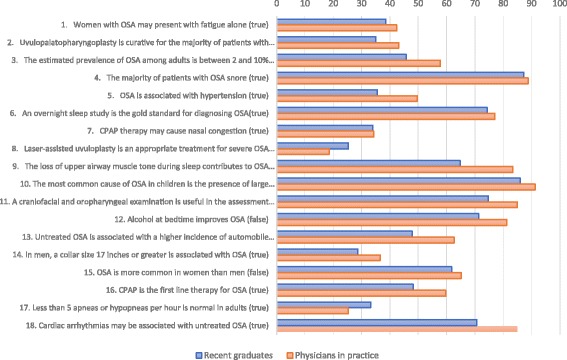
Percentage of participants reporting correct answers for each OSAKA comparing recent graduates with practicing physicians

None of the questions was answered correctly by all participants (Fig. 1). Overall, the item that was answered incorrectly by most respondents was, “Laser-assisted uvuloplasty is an appropriate treatment for severe OSA,” and the item answered correctly by most respondents was, “The most common cause of OSA in children is the presence of large tonsils and adenoids.” As shown in Table 1, the proportions of recent graduates answering each item correctly varied widely.

**Table 1:** Number (%) of participants reporting correct answers for each OSAKA item and results of unadjusted logistic regression models for each item comparing recent graduates with practicing physicians

Knowledge items	Recent graduates	Physicians in practice	Total	p*	Crude odds ratio (CI 95%)^a^
	*N = 265*	*N = 367*	*N = 656*		
	n (%)	n (%)	n (%)		
1. Women with OSA may present with fatigue alone (true)	102 (38.5)	156 (42.5)	258 (40.8)	0.311	0.85 (0.61–1.17)
2. Uvulopalatopharyngoplasty is curative for the majority of patients with OSA(false)	93 (35.1)	158 (43.1)	251 (39.7)	< 0.05	0.72 (0.52–0.99)
3. The estimated prevalence of OSA among adults is between 2 and 10% (true)	121 (45.7)	212 (57.8)	333 (52.7)	< 0.01	0.61 (0.45–0.85)
4. The majority of patients with OSA snore (true)	231 (87.2)	326 (88.8)	557 (88.1)	0.525	0.85 (0.53–1.39)
5. OSA is associated with hypertension (true)	94 (35.5)	182 (49.6)	276 (43.7)	< 0.001	0.56 (0.40–0.77)
6. An overnight sleep study is the gold standard for diagnosing OSA(true)	197 (74.3)	283 (77.1)	480 (76.0)	0.421	0.86 (0.60–1.24)
7. CPAP therapy may cause nasal congestion (true)	90 (34.0)	126 (34.3)	216 (34.2)	0.923	0.98 (0.71–1.37)
8. Laser-assisted uvuloplasty is an appropriate treatment for severe OSA (false)	67 (25.3)	68 (18.5)	135 (21.4)	< 0.05	1.49 (1.02–2.18)
9. The loss of upper airway muscle tone during sleep contributes to OSA (true)	172 (64.9)	306 (83.4)	478 (75.6)	< 0.001	0.37 (0.25–0.53)
10. The most common cause of OSA in children is the presence of large tonsils and adenoids (true)	228 (86.0)	335 (91.3)	563 (89.1)	< 0.05	0.59 (0.36–0.97)
11. A craniofacial and oropharyngeal examination is useful in the assessment of patients of large tonsils and adenoids (true)	198 (74.7)	312 (85.0)	510 (80.7)	< 0.01	0.52 (0.35–0.78)
12. Alcohol at bedtime improves OSA (false)	189 (71.3)	298 (81.2)	487 (77.1)	< 0.01	0.58 (0.40–0.84)
13. Untreated OSA is associated with a higher incidence of automobile crashes (true)	127 (47.9)	230 (62.7)	357 (56.5)	< 0.001	0.55 (0.40–0.75)
14. In men, a collar size 17 in. or greater is associated with OSA (true)	76 (28.7)	134 (36.5)	210 (33.2)	< 0.05	0.70 (0.50–0.98)
15. OSA is more common in women than men (false)	164 (61.9)	239 (65.1)	403 (63.8)	0.404	0.87 (0.63–1.21)
16. CPAP is the first line therapy for OSA (true)	128 (48.3)	219 (59.7)	347 (54.9)	< 0.01	0.63 (0.46–0.87)
17. Less than 5 apneas or hypopneas per hour is normal in adults (true)	88 (33.2)	93 (25.3)	181 (28.6)	< 0.05	1.47 (1.03–2.07)
18. Cardiac arrhythmias may be associated with untreated OSA (true)	187 (70.6)	312 (85.0)	499 (79.0)	< 0.001	0.42 (0.29–0.62)
Mean (SD) percentage of knowledge items answered correctly	53.5 (14.4)	60.4 (15.2)	57.5 (15.2)	< 0.001	

^a^Logistic regression, physicians in practice as reference group

*P for chi-square test comparisons between recent graduates and physicians in practice

**Fig. 2:**
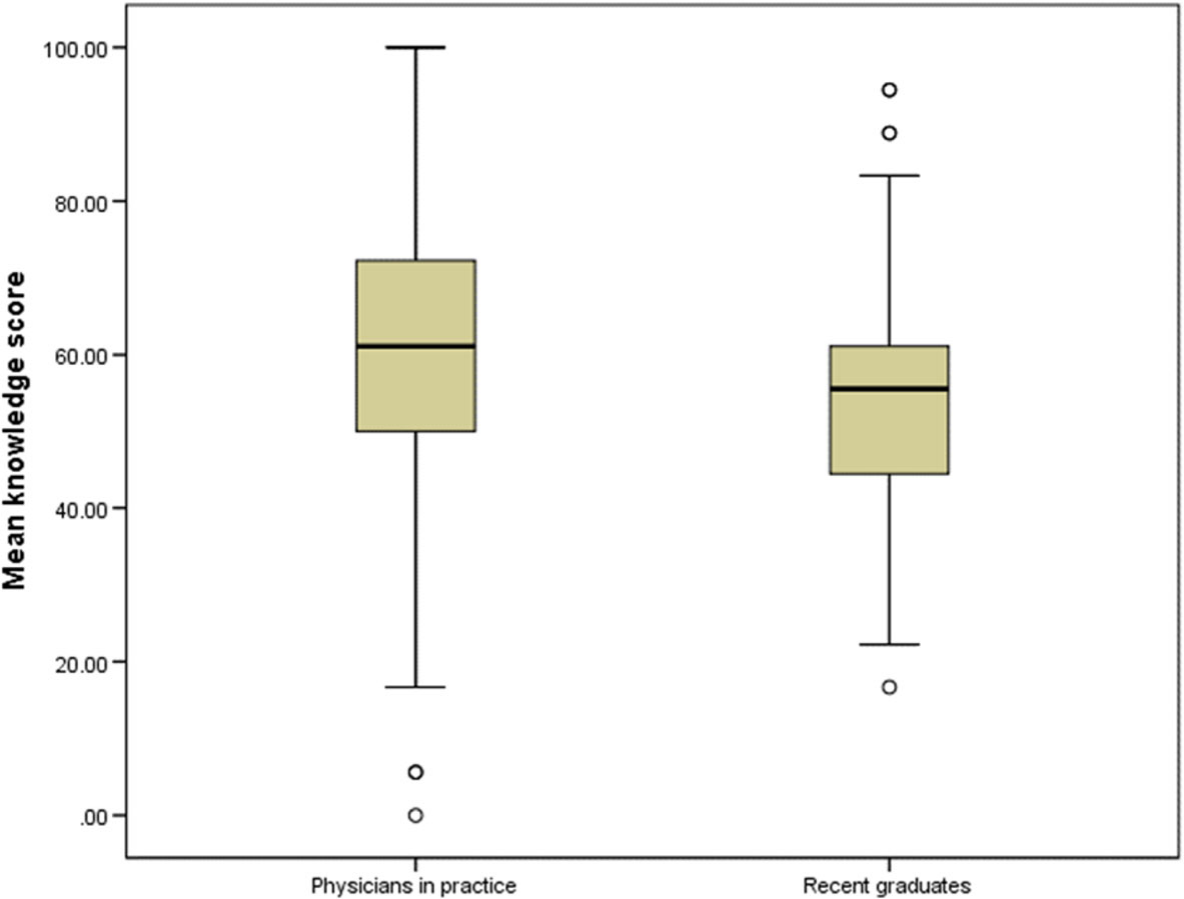
Boxplot of percentage of knowledge items answered correctly OSAKA comparing recent graduates with practicing Physicians

Eleven items measuring knowledge of diagnosis, treatment, and clinical outcomes of OSA were answered correctly by a greater proportion of practicing physicians compared with recent medical graduates (Fig. 1). The mean knowledge score was lower for recent graduates than for physicians in practice (53.5% vs. 60.4%; *p* < 0.001 (Fig. 2). Although low for both groups, a greater proportion of recent medical graduates than practicing physicians (33.2% vs 25.3%) knew that less than 5 apneas or hypopneas per hour is normal in adults (Table 1).

As shown in Table 2, a lower proportion of recent graduates from public compared with private universities correctly answered the questions about patients with OSA snore (item 4, 85% vs. 96.1%; *p* < 0.05) and a collar size in men greater than 17 in. being associated with OSA (item 14, 24.3% vs. 47.1%; *p* < 0.01). No significant differences were observed in mean knowledge scores by medical-school ownership (Table 2).

**Table 2:** Percentages of recent medical graduates answering each knowledge item correctly by university ownership

Knowledge items	University		
	*Private*	*Public*	p*
	*N = 51*	*N = 214*	
	n (%)	n (%)	
1. Women with OSA may present with fatigue alone	22 (43.1)	80 (37.4)	0.448
2. Uvulopalatopharyngoplasty is curative for the majority if patients with OSA	16 (31.4)	77 (36.0)	0.535
3. The estimated prevalence of OSA among adults is between 2 and 10%	21 (41.2)	100 (46.7)	0.474
4. The majority of patients with OSA snore	49 (96.1)	182 (85.0)	< 0.05
5. OSA is associated with hypertension	23 (45.1)	71 (33.2)	0.110
6. An overnight sleep study is the gold standard for diagnosing OSA	40 (78.4)	157 (73.4)	0.457
7. CPAP therapy may cause nasal congestion	12 (23.5)	78 (36.4)	0.080
8. Laser-assisted uvuloplasty is an appropriate treatment for severe OSA	9 (17.6)	58 (27.1)	0.163
9. The loss of upper airway muscle tone during sleep contributes to OSA	35 (68.6)	137 (64.0)	0.535
10. The most common cause of OSA in children is the presence of large tonsils and adenoids	43 (84.3)	185 (86.4)	0.693
11. A craniofacial and oropharyngeal examination is useful in the assessment of patients of large tonsils and adenoids	37 (72.5)	161 (75.2)	0.692
12. Alcohol at bedtime improves OSA	36 (70.6)	153 (71.5)	0.898
13. Untreated OSA is associated with a higher incidence of automobile crashes	29 (56.9)	98 (45.8)	0.155
14. In men, a collar size 17 in. or greater is associated with OSA	24 (47.1)	52 (24.3)	< 0.01
15. OSA is more common in women than men	28 (54.9)	136 (63.6)	0.253
16. CPAP is the first line therapy for OSA	29 (56.9)	99 (46.3)	0.173
17. Less than 5 apneas or hypopneas per hour is normal in adults	16 (31.4)	72 (33.6)	0.757
18. Cardiac arrhythmias may be associated with untreated OSA	34 (66.7)	153 (71.5)	0.497
Mean (SD) knowledge score (% of items answered correctly)	54.8 (13.0)	53.2 (14.7)	0.476

*Chi-square tests were used for comparisons between groups

### Importance of OSA and confidence in identifying and managing OSA

In our study, 44.2% of the students considered OSA to be important or extremely important as a clinical disorder. Similarly,40.4% considered identifying patients with OSA as important to extremely important. More than half (60.4%) of the respondents agreed or strongly agreed that they were confident in identifying patients with OSA. Almost half of participants (46.4%) agreed or strongly agreed that they were confident in their ability to manage OSA, but just 26.8% agreed or strongly agreed that they were confident in their ability to manage patients with CPAP therapy.

In general, physicians in practice attributed greater importance to OSA as clinical disorder and to identifying patients with OSA, and they reported greater confidence identifying patients at risk compared with recent graduates; but recent graduates reported greater confidence in managing patients with OSA and CPAP and on the confidence subscale score overall compared with physicians in practice (Table 3). No significant differences by gender, country, or university ownership were observed in attitudes among recent graduates (Table 4).

**Table 3:** Differences between recent graduates and physicians in practice in mean (SD) scores on the importance of OSA and confidence in identifying and managing patients with OSA

Attitude items	Total	Recent graduates	Physicians in practice	p*
	*N = 632*	*N = 265*	*N = 367*	
	Mean (SD)	Mean (SD)	Mean (SD)	
Importance of OSA as a clinical disorder	3.7 (0.9)	3.4 (0.8)	3.9 (0.8)	< 0.001
Important to identify patients with OSA	3.7 (0.9)	3.4 (0.8)	3.9 (0.8)	< 0.001
*Importance subscale score*	*3.7 (0.8)*	*3.4 (0.7)*	*3.9 (0.8)*	*< 0.001*
Confident identifying at-risk patients	3.6 (1.0)	3.5 (0.9)	3.7 (1.0)	0.024
Confident managing patients with OSA	3.1 (1.1)	3.3 (1.0)	2.9 (1.1)	< 0.001
Confident managing patients on CPAP	2.7 (1.1)	2.8 (1.0)	2.5 (1.1)	< 0.01
*Confidence subscale score*	*3.2 (0.9)*	*3.3 (0.9)*	*3.0 (0.9)*	*< 0.001*

*Tests of significance were one-way analyses of variance

**Table 4:** Differences in recent medical graduates’ mean (SD) scores on the importance of OSA and confidence in identifying and managing patients with OSA by university ownership

Attitude items	University		
	*Private*	*Public*	p*
	*N = 51*	*N = 214*	
	mean (SD)	mean (SD)	
Importance of OSA as a clinical disorder	3.3 (0.7)	3.4 (0.8)	0.829
Important to identify patients with OSA	3.2 (0.8)	3.4 (0.8)	0.211
*Importance subscale score*	*3.3 (0.7)*	*3.4 (0.7)*	*0.415*
Confident identifying at-risk patients	3.5 (1.1)	3.5 (0.9)	0.697
Confident managing patients with OSA	3.5 (0.9)	3.2 (1.0)	0.085
Confident managing patients on CPAP	2.9 (1.1)	2.8 (1.0)	0.484
*Confidence subscale score*	*3.5 (0.8)*	*3.3 (0.9)*	*0.147*

*Tests of significance were one-way analyses of variance

## Discussion

In our previous study we found no significant difference in total OSA knowledge by years in practice (< 5 vs > 5 years since graduation) and we hypothesized that this phenomenon may reflect a lack of adequate information regarding sleep disorders at the undergraduate and graduate medical education levels [9].

We found that mean knowledge about OSA was 53.5% among recent graduates, and there were no significant differences in mean knowledge scores by gender or university ownership. Our study demonstrated low levels of knowledge in recent medical graduates, similar to a previous study of Latin-American physicians already in practice [9].

Studies of recent medical graduates’ knowledge of OSA, attitudes toward OSA and its management are not common.

When we compared the present study with that of a previously published study using OSAKA questionnaire, we found that the level of knowledge among medical school graduates in our country was higher (53% compared with 43%) [18]. In Saudi Arabia [16], medical students were assessed about their knowledge about sleep using the Assessment of Sleep Knowledge in Medical Education (ASKME) Survey [16]. Only 4.6% of the respondents received a total score of ≥ 60% correct [16], showing very little knowledge about sleep and sleep disorders among medical students in selected schools in Saudi Arabia. We didn’t find differences between Public or Private Universities.

Thus, assessing sleep medicine knowledge among medical students and recent graduates, there is a clear need for sleep medicine curriculum to be introduced during medical school.

The absence of differences in total knowledge scores in our sample where participants were educated (University Public or Private) could be related to poor sleep curriculum during medical school regardless of school location. Multiple factors not measured in our study may be associated with a low knowledge score. Insufficient sleep curriculum could be one of the factors. In a multi-national study of medical schools [19], the authors identified prominent barriers to sleep education, including insufficient time devoted to sleep curriculum during the medical core program (32%) and lack of qualified staff (24%). The average amount of time devoted to sleep education was 2.5 h, and 27% of medical schools did not provide any education about sleep medicine. Similarly, a study in Italy reported a mean of 2.5 h [20], and a study in Saudi Arabia reported a mean of 2.6 h of sleep education during medical school [16]. The latter study reported two principal barriers to incorporate sleep medicine into the curriculum: the topic has a lower priority and time constraints do not allow more time for incorporating sleep medicine into the curriculum [16]. In our country from 1 to 4 h had been dedicated about sleep medicine education.

In addition, knowledge is not only dependent on the amount of time devoted to sleep medicine in the curriculum, but also on how the subject is taught. For example, education focused on recognizing symptoms of fatigue/sleepiness, understanding the basic physiology of sleep and sleep-wake rhythms, and providing a basic overview of common sleep disorders, with an emphasis on screening, diagnosis, and treatment of these disorders, was found to result in greater improvement in sleep knowledge than a sham module listing random sleep facts and trivia presented in slides [21].

Our study demonstrated low levels of knowledge in recent medical graduates, similar to a previous study of Latin-American physicians already in practice [9]. Patients with symptoms of OSA present to nearly all doctors, irrespective of specialty, and a basic knowledge of OSA is considered essential to identify these patients for appropriate referral and treatment. Moreover, in this current study, we directly compared the scores between recent graduates and physicians in practice and found that recent graduates were significantly less likely than physicians in practice to report correct answers to 10 of 18 knowledge items on the OSAKA. This lack of knowledge about the epidemiology, diagnosis, and treatment of OSA in recent medical graduates has implications for patient care during residency training, as lack of knowledge is an impediment to the timely diagnosis of OSA and referral of patients to a specialist, and can lead to an increased risk for later complications. For instance, patients frequently consult for hypertension, but our students demonstrated little knowledge about the relationship between OSA and hypertension. This observation is consistent with the findings reported in a study of medical students in Nigeria [18].

Furthermore, recent medical graduates who were surveyed did not respond correctly that cardiac arrhythmias may be associated with untreated OSA. Such beliefs could possible delay referral of patients to a specialist to initiate CPAP, which is the preferred treatment for patients with severe OSA.

Interestingly, recent medical graduates reported higher mean scores about confidence managing patients with OSA and CPAP than physicians in practice, which could reflect some self-protection or social desirability bias among recent graduates.

A lack of confidence in managing patients with sleep complaints and deficits in sleep knowledge are barriers to quality of care for OSA patients and can be attributed to a lack of exposure to sleep medicine in undergraduate and postgraduate medical education curricula [22].

But these findings are problematic in light of the low level of actual knowledge about OSA and its treatment that recent graduates reported. Given our findings in this study, medical schools are challenged to add greater emphasis on sleep medicine to their curricula to better enable medical students and graduates to detect OSA in patients and initiate treatment or to refer these patients to specialists for further examination and treatment.

The few hours of sleep medicine generally taught in medical schools around the world reflect a widespread need to enrich the medical-school curriculum with sleep medicine topics [18, 19]. Integrating more than a few hours of sleep medicine topics into the curriculum can better prepare students to identify and manage OSA during residency training, given the importance of the association between OSA and other life-threatening conditions [4, 5].

Future studies are needed to determine if incorporating OSA-focused educational interventios during medical school should help to improve recent medical graduates’ knowledge about OSA.

Our study had some limitations. First, this was a cross-sectional survey, and we cannot infer causation from any of the associations we observed. We also cannot generalize our results to other countries where medical education about OSA might differ in important ways. However, different studies also reported low levels of physicians’ knowledge about OSA diagnosis and treatment regardless of years of experience [12–17, 19–21].

However the strengths of our study include the use of a validated questionnaire [18, 19] and higher sample size. Future research could further validate the OSAKA by determining whether people with higher knowledge scores tend to appropriately refer patients at risk for OSA to get a sleep study. Improving medical school training on sleep disorders would likely increase the identification of persons at high risk of OSA and increase referral for consultation with expert sleep physicians [23].

Our next steps will be to focus future research on adding sleep medicine topics to the medical school curriculum and evaluating the impact of sleep medicine curricular change on physicians’ practice patterns and patient outcomes.

## Conclusions

Most of recent medical graduates in Ecuador did not know about OSA risk factors and appropriate management of OSA; not being able to recognize and appropriately refer patients at high risk of OSA for testing contributes to the under diagnosis of OSA and treatment. OSA-focused educational interventions during medical school should help to improve recent medical graduates’ knowledge about OSA, its diagnosis and referral for treatment. The addition of sleep medicine education to the current medical school curriculum, as well as a greater number of hours of medical students’ exposure to sleep education, could improve outcomes in patients with OSA.

## Abbreviations

ANOVA: Analyses of variance
ASKME: Assessment of Sleep Knowledge in Medical Education
CPAP: Continuous positive airway pressure
OSA: Obstructive Sleep Apnea
OSAKA: OSA Knowledge and Attitudes
PSG: Polysomnography
SD: Standard deviation
